# Meibography health score: a simple grading system for isotretinoin-induced meibography alterations on patients with acne vulgaris

**DOI:** 10.5935/0004-2749.2023-0150

**Published:** 2024-07-09

**Authors:** Fabio Mendonça Xavier Andrade, Flavio Hirai, Tais Hitomi Wakamatsu, Rebecca Ignacio Subira Medina, Denise de Freitas

**Affiliations:** 1 Department of Ophthalmology and Visual Sciences, Hospital São Paulo, Escola Paulista de Medicina, Universidade Federal de São Paulo, São Paulo, SP, Brazil; 2 Department of Dermatology, Hospital São Paulo, Escola Paulista de Medicina, Universidade Federal de São Paulo, São Paulo, SP, Brazil

**Keywords:** Isotretinoin, Meibography, Meibomian gland dysfunction, Radiologists, Ophthalmologists

## Abstract

**Purpose:**

To develop a simple, subjective, and reliable grading scale for isotretinoin-induced meibography changes.

**Methods:**

After analyzing meibography images obtained from systemic isotretinoin users, a grading scale was proposed and named “meibography health score.” The score ranged from 1 to 3, with decreasing gland reflectivity and identifiable margins. A total of 11 medical professionals were asked to grade 10 meibography images obtained from isotretinoin users using the proposed scale and were divided into three groups: (A) ophthalmologists with experience with meibography, (B) ophthalmologists with no experience with meibography, and (C) radiologists. The kappa statistic was determined to test interrater reliability.

**Results:**

The overall kappa was approximately 0.64. The kappa scores for Groups A, B, and C were 0.78, 0.59, and 0.90, respectively. Grade 2 had the lowest kappa scores (0.62, 0.35, and 0.82 for A, B, and C, respectively) and grade 3 the highest (0.78, 0.90, and 1.0 for A, B and C, respectively). Furthermore, Group C had the highest kappa scores and Group B the lowest.

**Conclusion:**

The meibography health score exhibited good interrater reliability, particularly in severe cases.

## INTRODUCTION

Over the past decades, meibography has proven to be an important tool for evaluating patients with dry eye and determining morphological changes accounting for meibomian gland dysfunction (MGD). This technique involves capturing an image of the meibomian glands (MG) using infrared light aimed at the everted tarsal plates^(1,2,3)^.

The use of systemic isotretinoin is widely known to cause MGD, but a variety of other conditions and/or diseases can induce MGD-related morphological changes in the MG. Examples of these factors include rosacea, contact lens wear, chemotherapy, radiotherapy, and even aging^(4)^.

Regardless of the cause, different grading scales and scores have been proposed and used to describe meibography changes, most of which analyze the area of MG loss or absence^(3,4,5,6,7,8)^.

Isotretinoin is a retinoid commonly used to treat acne, and its main effects on MG are atrophy and density reduction. Notably, these effects are diffuse and not focal; thus, dropout and shortening of the glands are not necessarily seen on meibography. Instead, a uniform loss of contrast and margin delineation is characteristically observed in these patients, and at present, no grading scale contemplates these changes. To date, no grading scales that express the unique pattern of systemic isotretinoin-induced meibography alterations have been developed^(9,10,11)^.

This study aimed to propose a new and simple grading scale for isotretinoin-induced meibography changes and to evaluate its reliability among different raters.

## METHODS

After signed informed consent, the upper tarsus of 28 patients with acne vulgaris receiving treatment for the first time with oral isotretinoin (0.5 mg/kg) were evaluated through noncontact meibography using Oculus Keratograph 5M (Oculus, Wetzlar, Germany). The evaluation was conducted before and after 16 weeks of treatment. A grading scale of meibography changes was then designed and named “meibography health score (MHS)”. The upper tarsus was selected because abnormal morphological features of MG more commonly exist in the upper lid^(1)^.

**Figure 1:**
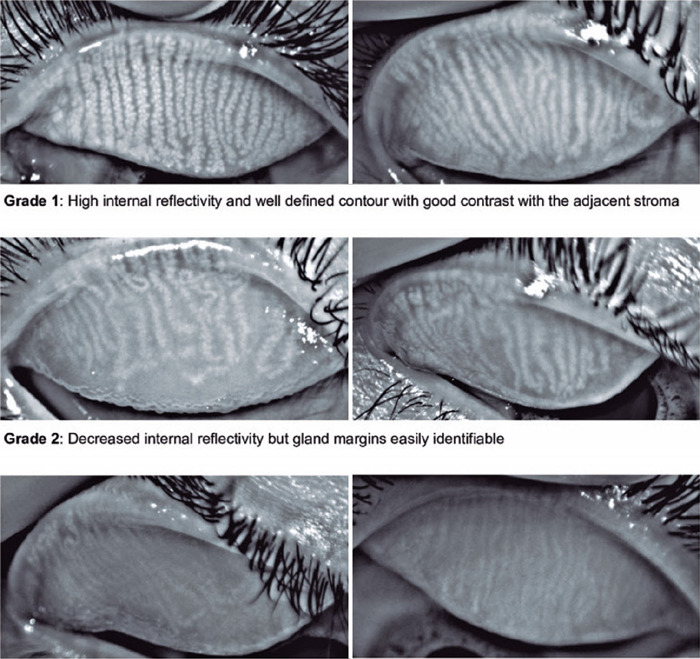
Meibography health score.

The proposed score ranged from 1 to 3 according to the MG morphology regarding gland internal reflectivity and gland margin delineation (Figure 1). Grade 1 represented a gland without alterations and was determined by high internal reflectivity and a well-defined contour exhibiting good contrast with the adjacent stroma. In grade 2, a decrease in internal reflectivity was observed (light gray), but the gland margins remained easily identifiable (if desired, the gland width could still be easily measured). Grade 3 was determined by low internal reflectivity and unclear gland margins. In this grade, the glands were very pale, almost the same grayscale level as the adjacent stroma, making it very difficult to measure their width. The rationale for creating a three-category scale was observational. Most of the patients exhibited a grade 1 score before starting isotretinoin treatment, and grades 2 and 3 reflected the range of postmedication atrophy that was observed.

**Figure 2:**
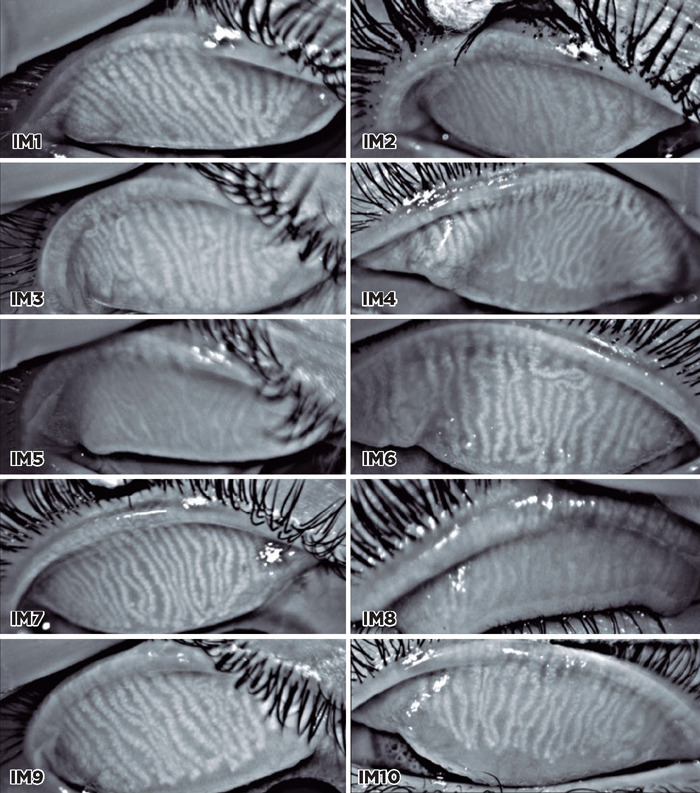
Selected meibography images of systemic isotretinoin users.

A total of 10 meibography images were selected, and 11 viewers were asked to grade the images using the proposed scale (Figure 2). The viewers were divided into three groups: (A) ophthalmologists with experience with meibography, (B) ophthalmologists with no experience with meibography, and (C) radiologists. Radiologists were selected as they use different and new grading scales every day. The kappa statistic was determined to test interrater reliability for all participants and between the groups using software StataCorp (Stata Statistical Software: Release 14. College Station, TX). A kappa score of 0.41-0.60 indicated low reliability; 0.61-0.80, moderate; and >0.81, high.

## RESULTS

A total of 11 medical professionals participated and were divided into Group A (n=3), Group B (n=5), and Group C (n=3). Their answers are shown in table 1.

**Table 1:** Researchers’ answers

		IM1	IM2	IM3	IM4	IM5	IM6	IM7	IM8	IM9	IM10
**Group A**	**R1**	1	3	1	2	3	1	1	3	1	2
	**R2**	1	2	1	2	3	1	1	3	1	2
	**R3**	1	2	1	2	3	1	1	3	1	1
**Group B**	**R4**	2	3	2	2	3	1	1	3	1	1
	**R5**	2	3	2	3	3	1	1	3	1	1
	**R6**	1	3	2	1	3	1	1	3	1	2
	**R7**	1	3	2	2	3	1	1	3	1	2
	**R8**	1	3	2	2	3	2	1	3	2	1
**Group C**	**R9**	1	3	2	2	3	1	1	3	1	1
	**R10**	1	3	2	2	3	1	1	3	1	2
	**R11**	1	3	2	2	3	1	1	3	1	2

R= researcher; IM= image. R1-R3= experienced ophthalmologists; R4-R8= inexperienced ophthalmologists; R9-R11= radiologists.

When analyzing all participants, the kappa scores were 0.62, 0.40, and 0.87 for grades 1, 2, and 3, respectively. The overall kappa was 0.64.

For Group A, the kappa scores were 0.86, 0.62, and 0.81 for grades 1, 2, and 3, respectively. The overall kappa was 0.78.

For Group B, the kappa scores were 0.50, 0.35, and 0.90 for grades 1, 2, and 3, respectively. The overall kappa was 0.59.

For Group C, the kappa scores were 0.86, 0.82, and 1.0 for grades 1, 2, and 3, respectively. The overall kappa was 0.90.

The results are summarized in table 2.

**Table 2:** Kappa score results

	Grade 1	Grade 2	Grade 3	General
All participants	0.62	0.40	0.87	0.63
Group A	0.86	0.62	0.81	0.78
Group B	0.50	0.35	0.90	0.59
Group C	0.86	0.82	1.00	0.89

Group A= experienced ophthalmologists; Group B= inexperienced ophthalmologists; Group C= radiologists.

## DISCUSSION

Noncontact meibography can evaluate the morphology of MG and provide valuable information for different ocular surface diseases or predisposing conditions, including standard MGD, use of topical glaucoma medication, contact lens wear, and ocular rosacea^(12,13,14,15)^.

In 2008, Arita et al. described meibography changes observed with aging and developed the *meiboscore*, a grading system based on areas of gland loss, or dropout, which has been used in many studies after the publication, including automated analysis^(3,16)^.

In the meibography of oral isotretinoin users, it was observed that the glands underwent a uniform and diffuse shrinkage, associated with reflectivity loss and margin delineation, without necessarily increasing dropout areas. It is possible to see these changes in images from other studies^(10,17)^.

Other investigators have created tools to demonstrate MG atrophy without dropout. In 2019, the *vagueness value* was developed by Yin et al., combining the use of a software and a manual algorithm to express the difference in contrast between the gland and the tarsal plate^(18)^. In addition, Yeh et al. used a software to determine contrast in meibography images through pixel intensity measurements^(19)^. Although useful in research, these measurements made using complex algorithms and specialized software are not practical. An observational and simple score for describing isotretinoin-induced diffuse MG atrophy can be very useful in daily practice if sufficient interrater reliability is achieved.

In our study, kappa analysis revealed that overall, the interrater reliability was moderate (k=0.64). Grade 3 was easier to match between readers, with a kappa score of 0.87, and grade 2 had the lowest kappa score (0.40). These results are understandable as changes between grades 1 and 2 are more subtle, and the reader must pay attention to the reflectivity of the gland, which is lower in grade 2 but without complete loss of margin delineation as observed in grade 3. This can also explain why radiologists, as professionals accustomed to details, exhibited the highest interrater reliability, even without experience with meibography. Furthermore, as our results indicated better reliability in Group A than in Group B, we should expect ophthalmologists to improve their reliability as they gain sufficient experience with meibography.

In conclusion, the MHS was successfully developed and has the potential to be an extremely useful tool for grading meibography alterations in systemic isotretinoin users, with good interrater reliability, particularly in severe cases. A prospective study using the proposed scale should be conducted to determine if there is a correlation among MHS stages, medication dose, and clinical symptoms.
